# Supplementary material for: Recombination between the Fostera MLV-like Strain and the Strain Belonging to Lineage 1 of Porcine Reproductive and Respiratory Syndrome Virus in Korea
Source: Viruses. 2022 May 26;14(6):1153. doi: 10.3390/v14061153 (PMC9229315; doi:10.3390/v14061153)
Supplement: Supplementary file 1 [file viruses-14-01153-s001.zip › viruses-1706369-supplementary.pdf]

viruses

MDPI

Table S1. Identities of complete genomic sequence of 20D160-1 and 21R2-63-1 isolates to other PRRSV reference strains.

	**Pairwise % Identity to 20D160-1 (nt)**										
	**VR2332**	**LMY**	**P129**	**TJ**	**CG**	**SDSU73**	**NADC30**	**NADC31**	**JB15-N-P31-GB**	**CA-2**	**KNU-1902**
	Lineage 5			Lineage 8			Lineage 1			Kor C	
ORF1a	87.97	85.39	98.94	89.32	88.91	93.02	79.51	77.55	77.44	77.96	75.82
ORF1b	92.25	90.42	99.33	92.70	92.44	94.72	87.92	85.83	85.79	87.22	86.55
ORF2	85.58	87.68	88.44	85.57	85.41	85.98	82.00	83.41	81.62	84.63	95.15
ORF3	82.97	81.10	82.72	80.62	80.62	81.91	77.60	78.13	75.16	88.54	96.64
ORF4	85.29	85.78	88.06	87.17	86.71	86.26	85.26	81.91	83.05	93.54	96.75
ORF5	89.79	86.66	97.97	92.37	92.18	93.32	84.24	84.24	83.79	83.17	77.50
ORF6	96.27	94.23	99.62	97.66	97.66	99.23	87.98	92.46	88.64	92.24	89.36
ORF7	93.44	91.28	99.46	94.94	94.94	94.35	91.26	88.71	89.04	92.25	88.70
	Pairwise % Identity to 21R2-63-1 (nt)										
	VR2332	LMY	P129	TJ	CG	SDSU73	NADC30	NADC31	JB15-N-P31-GB	CA-2	KNU-1902
	Lineage 5			Lineage 8			Lineage 1			Kor C	
ORF1a	76.27	74.64	75.03	72.86	73.19	74.71	90.00	78.82	92.32	75.66	73.29
ORF1b	85.73	83.71	88.59	85.85	85.62	87.57	91.55	85.64	92.07	83.30	82.35
ORF2	84.71	83.86	85.14	83.09	82.25	84.04	93.56	83.20	96.39	81.30	81.70
ORF3	80.07	80.50	79.87	79.30	78.61	80.29	93.10	81.68	95.11	78.86	76.56
ORF4	82.87	84.39	83.47	81.49	81.00	84.17	93.74	87.66	94.97	83.44	82.96
ORF5	80.32	79.39	81.46	80.01	80.33	80.86	90.84	90.84	92.39	81.98	83.10
ORF6	89.76	90.20	89.08	89.33	89.78	88.88	95.65	90.30	96.46	87.36	87.60
ORF7	89.76	88.39	89.03	88.38	89.03	88.39	94.96	88.03	96.69	90.05	85.24

Table S2. Recombination breakpoints of 20D160-1 and 21R2-63-1 with associated parental strains detected by RDP4 software.

**Isolates**	**Method**	**Breakpoint Position (nt) ***		**Major Parent**	**Minor Parent**	***p*-Value**
		**Beginning**	**Ending**			
20D160-1	RDP	11,990	13,563	P129	KNU-1902	2.806×10^{-88}
	GENECONV					9.770×10^{-93}
	BootScan					2.169×10^{-136}
	MaxChi					7.207×10^{-32}
	Chimaera					2.351×10^{-31}
	SiScan					5.933×10^{-28}
	3Seq					6.661×10^{-15}
21R2-63-1	RDP	7,654	8,599	NADC30	P129	6.080×10^{-29}
	GENECONV					7.742×10^{-51}
	BootScan					2.051×10^{-63}
	MaxChi					4.893×10^{-18}
	Chimaera					2.792×10^{-19}
	SiScan					1.321×10^{-20}
	3Seq					2.220×10^{-15}

* Nucleotide (nt) position based on VR2332 strain.
